# A Basis for Rapid Clearance of Circulating Ring-Stage Malaria Parasites by the Spiroindolone KAE609

**DOI:** 10.1093/infdis/jiv358

**Published:** 2015-07-01

**Authors:** Rou Zhang, Rossarin Suwanarusk, Benoit Malleret, Brian M. Cooke, Francois Nosten, Yee-Ling Lau, Ming Dao, Chwee Teck Lim, Laurent Renia, Kevin Shyong Wei Tan, Bruce Russell

**Affiliations:** 1 Department of Microbiology, Yong Loo Lin School of Medicine; 2 Biomedical Engineering, National University of Singapore; 3 Singapore Immunology Network (SIgN), Agency for Science Technology and Research (A*STAR), Biopolis; 4 Department of Microbiology, Monash University, Melbourne, Victoria, Australia; 5 Shoklo Malaria Research Unit, Mae Sot, Tak; 6 Faculty of Tropical Medicine, Mahidol University, Bangkok, Thailand; 7 Centre for Tropical Medicine, Nuffield Department of Clinical Medicine, University of Oxford, United Kingdom; 8 Department of Parasitology, Faculty of Medicine, University of Malaya, Kuala Lumpur; 9 Department of Materials Science and Engineering, Massachusetts Institute of Technology, Cambridge

**Keywords:** *Plasmodium vivax*, *Plasmodium falciparum*, malaria, red blood cell, spiroindolones, KAE609

## Abstract

Recent clinical trials revealed a surprisingly rapid clearance of red blood cells (RBCs) infected with malaria parasites by the spiroindolone KAE609. Here, we show that ring-stage parasite–infected RBCs exposed to KAE609 become spherical and rigid, probably through osmotic dysregulation consequent to the disruption of the parasite's sodium efflux pump (adenosine triphosphate 4). We also show that this peculiar drug effect is likely to cause accelerated splenic clearance of the rheologically impaired *Plasmodium vivax*– and *Plasmodium falciparum*–infected RBCs.

The emergence of *Plasmodium* parasites with increased tolerance to artemisinins, the only remaining globally effective antimalaria drug, poses a serious threat to malaria control. The recently discovered spiroindolone class of compounds show promise as effective antimalarials [1]. In human phase II clinical trials, the most advanced of these, KAE609 (cipargamin; formerly NITD609; Novartis Institute for Tropical Diseases), proved to be highly effective in treating adults with uncomplicated *Plasmodium vivax* or *Plasmodium falciparum* malaria [2]. Of particular interest was the unexpectedly rapid clearance of red blood cells (RBCs) infected with early blood-stage (ring-stage) parasites, with median half-lives for parasite clearance of <1 hour (Figure 1*A*) [2], which compares favorably with a geometric mean of 2.6 hours measured for artesunate treatment of uncomplicated falciparum malaria in 2001 on the Thai-Myanmar border [3].

**Figure 1.**
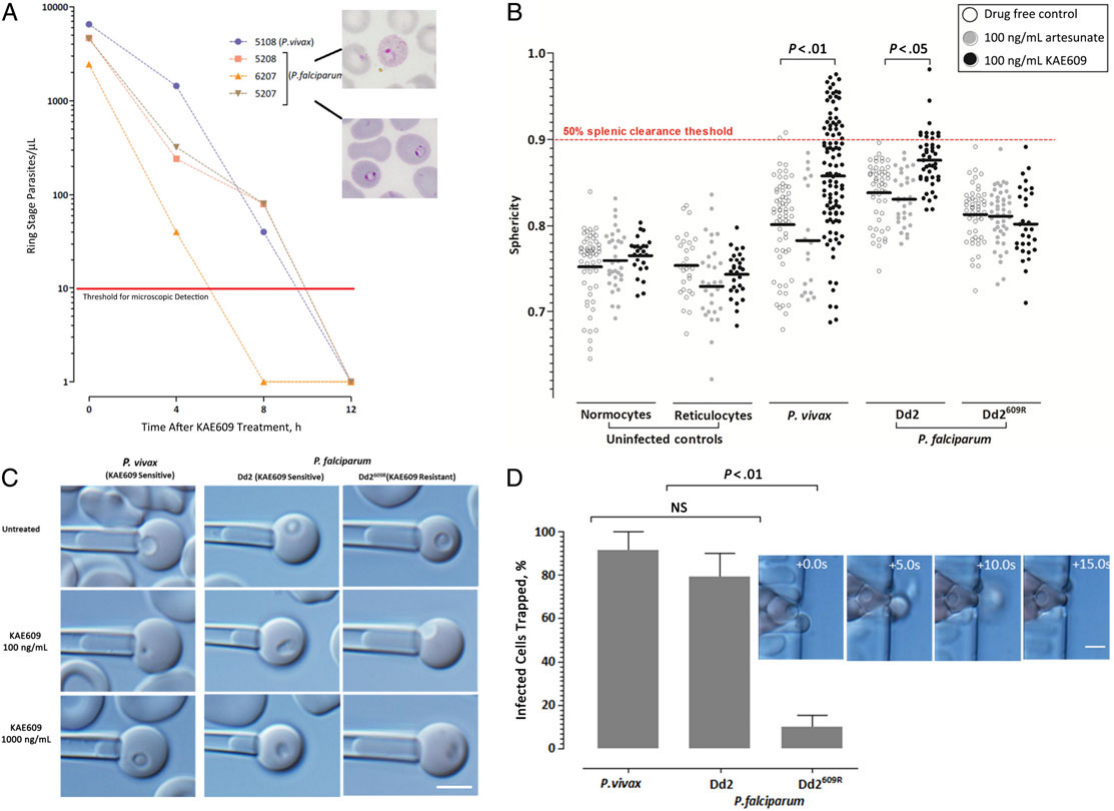
Rapid disappearance of circulating *Plasmodium vivax* and *Plasmodium falciparum* ring-stage parasites in KAE609-treated patients with malaria is due to significant changes in the morphological and rheological properties of the infected red blood cells (RBCs). *A,* Giemsa-stained thin blood smears from a recent phase II clinical study [2] were reread by 2 expert microscopists to determine the unreported stage of parasite development present (at admission [hour 0] and 3 subsequent sampling times). This new analysis revealed that in all 4 infected individuals (1 patient with *P. vivax* and 3 with *P. falciparum* malaria), parasites were at an synchronous young ring stage (approximately 4–8 hours after RBC invasion). No gametocytes were detected, except in the patient with *P. vivax* malaria (approximately 40 gametocytes per microliter). Four hours after treatment, KAE609 resulted in 78%–94% clearance of the parasites, including 100% of all *P. vivax* gametocytes. No parasites could be detected 12 hours after treatment (red line indicates microscopic threshold of detection). Note the Giemsa-stained thin film images of representative *P. vivax* (*upper inset*) and *P. falciparum* (*lower inset*) ring-stage–infected RBCs present in the samples. *B,* Micropipette aspiration was used to determine the sphericity (sphericity index [SI] of 1 indicates a perfect sphere) of RBCs infected with ring-stage malaria parasites that were either sensitive (*P. vivax* [6 isolates] and *P. falciparum* Dd2 [3 independent trials]) or resistant (*P. falciparum* Dd2^R609^ [6 independent trials]) to spiroindolone (KAE609) and/or artesunate after 2 hours of treatment with 100 ng/mL of these drugs. The sphericity of infected RBCs of *P. vivax* (median SI, 0.801–0.858; *P* < .01) and Dd2 (median SI, 0.838–0.876; *P* < .05) treated with KAE609 significantly increased relative to drug-free controls. However, no change was detected in *P. falciparum* Dd2^R609^ (median SI, 0.813–0.811; *P* = .39). Red dotted line (extrapolated from ex vivo human spleen data [9]) indicates the threshold sphericity (SI >0.9) at which ≥50% of RBCs will be cleared. Only *P. vivax–* and Dd2 *P. falciparum*–infected RBCs treated with KAE609 show an appreciable proportion (>20% of the cell population) of cells with an SI >0.9. Importantly. none of the drug treatments have any effect on the SI of uninfected RBCs. Each circle represents a single infected RBC. *C,* The effect of KAE609 on the deformability of infected RBCs, is illustrated by the length of the aspirated cell at an aspiration pressure of 588.6 Pa (ie, infected RBCs with a lower shear modulus/higher deformability will have a longer aspirated tongue). Thus, in the case of the sensitive *P. vivax*– and Dd2-infected RBCs, treatment with 100 and 1000 ng/mL KAE609 results in discernibly shorter aspirated cell lengths compared with the KAE609-resistant clone Dd2^R609^. *D*, Increase in sphericity of KAE609-treated *P. vivax*– and *P. falciparum* Dd2–infected RBCs resulted in a significantly (*P* < .01) higher median percentage of infected RBC (normalized for parasitemia) trapping in 2-µm microfluidic restrictions (which model the splenic sinusoidal slits), compared with the KAE609-resistant clone (Dd2^R609^). The inset to the left of the bar graph shows a 15-second sequence (5-second frames) of a drug-affected Dd2 trapped in the 2-µm gap (almost all infected RBCs remain trapped for the duration [5–15 minutes] of the experiment). (For the video version of this inset, see Supplementary Figure 3.)

Given that KAE609 and artemisinins have distinct mechanisms of action, we have explored and elucidated the nature of the rapid clearance. We hypothesized that KAE609, known to be a potent adenosine triphosphatase 4 (ATPase4; malaria parasite sodium efflux pump adenosine triphosphate 4 [ATP4]) inhibitor [4], is likely to alter the biochemical and rheological properties of parasite-infected RBCs by dysregulating the parasite's ionic homeostasis.

## METHODS

### Reexamination of Slides From a Clinical Study

We obtained approval to reexamine slides from the 4 patients with malaria (the entire group of patients enrolled in the clinics associated with the Shoklo Malaria Research Unit from a phase II, open-label study of KAE609 [2]). Although the original study reports parasitemia, it does not provide any data on the stage of the parasites. Because the ring-stage parasitemia is central to this study, it was important to stage the parasites carefully. We used 2 expert microscopists to examine and quantify the number of parasites by stage per 1000 white blood cells in the thick film and per 8000 RBCs in the thin film (Figure 1*A*).

The original study and the collection of clinical isolates (*P. vivax*) used in this study was approved by the ethics committees of the Faculty of Tropical Medicine, Mahidol University, the Thai Ministry of Public Health, the Institute for the Development of Human Research Protection, and the Oxford Tropical Research Ethics Committee (OxTREC).

### Cells and Reagents

We used the *P. falciparum* Dd2 strain, a drug-resistant Dd2^609R^ line derived from it (a gift from the Novartis Institute of Tropical Diseases), and *P. vivax* from 10 clinical isolates (>80% ring stage) collected from individuals attending health clinics of the Shoklo Malaria Research Unit in Thailand (collected in 5-mL lithium-heparinized tubes and sent to the laboratory within 6 hours), under the ethical guidelines OxTREC 58-09 and 04-10. JC-1 and Hoechst 33 342 were purchased from Life Technologies, ionomycin was purchased from Invitrogen, and the Annexin V:FITC Apoptosis Detection kit was purchased from BD Biosciences.

### Short-Term Treatment of Infected RBCs With KAE609 and Artesunate

Stage-synchronous *P. vivax–* or *P. falciparum–*infected RBCs (>80% ring stage <8 hours after invasion) were maintained in 200 µL of McCoy 5A medium (supplemented with 2.4 g/L D-glucose and 20% heat-inactivated human AB serum), at a 2% hematocrit. This blood medium mixture was treated with artesunate at 100 ng/mL and KAE609 at 100 ng/mL, and 1000 ng/mL and then incubated in of McCoy medium for 2 hours in a gas mix incubator (5% carbon dioxide, 5% oxygen, and 90% nitrogen) at 37.5°C before assessment of its morphological and biomechanical changes. An additional control group was prepared to assess the properties of untreated infected RBCs [5]. The sphericity of uninfected reticulocytes and normocytes from control and treated isolates was also determined.

### Micropipette Aspiration and RBC Sphericity Measurement

The sphericity of infected RBCs was measured using the method of Waugh et al [6]. Cell suspensions were viewed under a ×100 oil immersion objective (Olympus IX73). A borosilicate micropipette with an inner diameter of 1.5 ± 0.2 µm was used to aspirate the infected RBCs at a negative pressure of 588.6 Pa (6 cm H_2_O). Cell deformation was recorded using a Dual CCD Digital Camera DP80 (Olympus) and analyzed using cellSens Dimension software (version 1.9).

### Microfluidics

Polydimethylsiloxane microfluidic flow chambers with 2-µm openings were used for ring-stage–infected RBCs. Channels were prefilled and incubated with ×1 phosphate-buffered saline with 1% bovine serum albumin for 1 hour to prevent RBC adhesion to polydimethylsiloxane before experiments. After incubation with KAE609, 1 µL of packed RBCs (containing 5% *P. falciparum*–infected RBCs or <1% *P. vivax*–infected RBCs) was injected into the microfluidic channels. Cells were pushed through the channel at a constant pressure gradient of 0.1 Pa/µm.

### JC1 Cell Viability Assay

We used methods derived from Ch'ng et al [7] and Pasini et al [8]. Briefly, after incubation with KAE609, *P. falciparum*–infected RBCs were resuspended in Roswell Park Memorial Institute 1640 medium, and *P. vivax*–infected RBCs were resuspended in McCoy's medium. JC1 (6 µmol/L) and Hoechst 33 342 (1 µg/mL) were added. The cell suspension was incubated at 37°C for 30 minutes, and then washed with ×1 phosphate-buffered saline. Fluorescence images were captured under a ×100 oil immersion objective (Olympus IX73) with additional ×2 tube magnification. Images were analyzed using cellSens – Dimensions software.

### Annexin V Apoptosis Assay

KAE609-treated infected RBCs were resuspended in 200 µL of ×1 annexin V binding buffer containing 10 µL of annexin V–fluorescein isothiocyanate and 1 µg/mL Hoechst 33 342. Cells were incubated at room temperature for 15 minutes in the dark and then washed with ×1 binding buffer. Samples were analyzed using an LSR II flow cytometer (Becton Dickinson). FlowJo software (version 10.0.7) (Tree Star) was used for flow cytometry analyses. Phosphatidylserine expression was also confirmed using fluorescence microscopy (Olympus IX73). A phosphatidylserine-positive control group was prepared by incubating uninfected RBCs in Ringer solution (125 mmol/L sodium chloride, 5 mmol/L potassium chloride, 1 mmol/L magnesium sulfate, 32 mmol/L HEPES, 5 mmol/L D-(+)-glucose, and 1 mmol/L calcium chloride, pH adjusted to 7.4) with 1 µmol/L ionomycin for 30 minutes at 37°C, followed by annexin V–fluorescein isothiocyanate staining.

### Sequencing PfATP4 and PvATP4

To determine allelic differences in PF3D7_1211900 (PfATP4) and PVX_084625 (PvATP4), the gene was subjected to polymerase chain reaction (PCR) amplification with Phusion DNA polymerase (Thermo Scientific) from genomic DNA, using primers and master mix, as detailed in Supplementary Figure 1. PCR products were purified with the QIAquick PCR purification kit according to the manufacturer's instructions and sequenced with AITbiotech sequencing. Sequences were aligned and analyzed using Lasergene SeqMan Pro software (version 9.1.0) (DNASTAR).

### Statistical Analysis

Nonparametric analysis of data in Figure 1*A* (Mann–Whitney) and Figure 1*D* (Kruskal–Wallis with Dunn's post-hoc tests) was performed using GraphPad Prism 6 software.

## RESULTS

To examine the effect of KAE609 on the rheological properties of infected RBCs, we obtained ring-stage parasites collected from individuals infected with *P. vivax* (n = 10) or from in vitro cultures of *P. falciparum* (line Dd2 and the KAE609-resistant counterpart, Dd2^609R^), which were then exposed for 2 hours to KAE609 or to artesunate at a concentration equivalent to that commonly observed therapeutically (100 ng/mL) [2]. The resulting alterations in sphericity and rigidity were then measured by micropipette aspiration of subsets of single RBCs (infected and uninfected). The sphericity of control RBCs (normocytes and reticulocytes) was not significantly affected by exposure to either drug (Figure 1*B*) [9]. Parasite–infected RBCs were also unaffected by exposure to artesunate. By contrast, the drug-sensitive *P. vivax*–infected and *P. falciparum* Dd2–infected RBCs showed a marked increase in sphericity after exposure to KAE609, a phenomenon not observed for the KAE609-resistant Dd2^609R^ parasites (Figure 1*B* and 1*C*). Amplification and sequencing confirmed that Dd2^609R^ harbors 2 of the 3 key *pfATP4* mutations associated with resistance [4], and all of the *P. vivax* isolates used in this study carried the wild-type genotype at the orthologous ATPase loci (Supplementary Figure 1).

The increase in sphericity is associated with a decrease in the deformability (increase in rigidity) of the RBCs (infected or uninfected), as evidenced by a shorter tongue of the RBC that is aspirated in the micropipette at a constant negative pressure of 588.6 Pa (6 cm H_2_O) (Figure 1*C*). We then ascertained whether the demonstrable increase in the sphericity and rigidity of the parasite-infected RBCs subsequent to exposure to KAE609 were sufficient to result in their retention (and subsequent clearance) in the spleen. Thus, control and KAE609-treated infected RBCs were perfused through an in vitro microfluidic device that models the sinusoids in the spleen [10]. The proportion of KAE609-treated infected RBCs that remained trapped in the 2-µm restrictions of the flow channels was significantly higher than that observed for both the untreated RBC controls and the drug-exposed KAE609-resistant Dd2^609R^ *P. falciparum* clone (Figure 1*D*).

## DISCUSSION

It should be noted that RBCs infected with young, ring-stage parasites, during their first 6 hours of intraerythrocytic development, do not alter measurably the rheological properties of normal infected RBCs [11]. Thus, infected RBCs containing ring-stage parasites would not be expected to be retained by the mechanical barriers imposed by the splenic sinusoids (approximately 2 µm in diameter). On the other hand, using and elegant and unusual system of perfused ex vivo human spleens, Safeukui et al [9] demonstrated that at least half of the RBCs whose sphericity index exceeds 0.90 are retained in the splenic sinusoids. Given our observations here that exposure to KAE609 increased the sphericity of approximately 24%–31% of ring-stage–infected RBCs beyond a sphericity index of 0.9 (Figure 1*B*), making them grossly poorly deformable, implies that a significant proportion would be unable to traverse the splenic sinusoids and then be trapped and destroyed by splenic phagocytes. This phenomenon is unlikely to occur with infected RBCs exposed to high levels of artesunate because this drug did alter RBC sphericity (Figure 1*B*).

**Figure 2.**
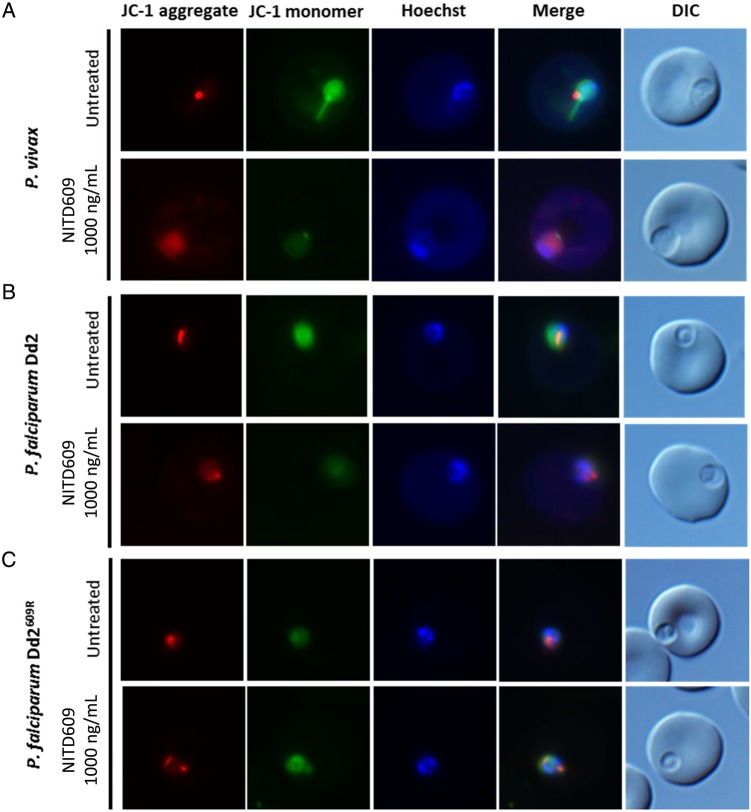
To investigate whether KAE609 treatment is lethal for parasites, we used JC1 (a mitochondrial probe that allows visualization of live [red staining] and/or dead [absence of red staining] parasites) as a marker of parasite viability. With KAE609 at 100 ng/mL, we saw no evidence of cell death (data not shown). *A, B*, However, after 2 hours of KAE609 treatment at 1000 ng/mL (approximately maximum concentration 2–4 hours after oral administration of 30 mg), *Plasmodium vivax* and Dd2 parasites showed clear signs of death with the loss and dispersal of JC1 monomer and aggregate staining. *C*, As expected, the *Plasmodium falciparum* Dd2^R609^ showed no sign of cell death. Abbreviation: DIC, differential interference contrast.

This rapid perturbation by KAE609 of the rheological properties of parasite-infected RBCs could be due to ATP4-mediated osmotic dysregulation and/or to the induction parasite cell death and eryptosis. JC1 (a mitochondrial probe that permits visual discrimination of live and dead parasites) and annexin V (as a measure of phosphatidylserine exposure) were used to assess parasite and RBC viability [7, 12]. Whereas *P. vivax*– and *P. falciparum* Dd2–infected RBCs exposed to 100 ng/mL KAE609 remained viable for a period of 2–4 hours (data not shown), those exposed to 1000 ng/mL of the drug showed clear signs of mitochondrial stress and structural disruption and often displayed ruptured parasitophorous vacuoles, with parasite contents spilling into the RBC cytoplasm (Figure 2*A* and 2*B*). The KAE609-resistant Dd2^609R^ line remained unaffected by exposure at the higher concentration (Figure 2*C*). None of the treatments led to abnormal exposure of phosphatidylserine on the RBC surface (Supplementary Figure 2*A* and 2*B*), although KAE609-treated parasites did show other signs of eryptosis, including cell shrinkage and membrane blebbing after prolonged exposure to 1000 ng/mL KAE609.

To some extent, the phenomenon we document here, namely, alteration of the physical properties of infected RBCs, is not unique to KAE609. Indeed, 2 other preclinical lead compounds that affect the parasites' ATP4 enzyme, a pyrazoleamide, PA21A092 [13], and a dihydroisoquinolone, (+)-SJ733 [14], were reported to induce swelling of infected RBCs, with a suggestion that this could be due to the induction of eryptosis/senescence for (+)-SJ733. Nonetheless, the drug-induced rheological alterations caused by (+)-SJ733 (observations on PA21A092 in this context were limited) differed from those we have made with KAE609 in that they were made using infected RBCs with maturing trophozoites in which significant biomechanical changes became evident 7–24 hours after compound exposure [14]. Moreover, the overall parasite clearance rates observed in the humanized mouse model seemed slower that those measured for KAE609 in the clinical trial [2].

Ultimately, whereas antimalaria drugs, including KAE609, directly kill the parasite by inhibiting its key metabolic pathways or through oxidative damage, KAE609 is the first clinically tested drug that exerts part of its antimalaria activity through the biochemical disruption of the infected RBC. This dual killing mechanism seems to be unique to the spiroindolone class of drugs and probably contributes in large part to the remarkably fast parasite clearance observed, which exceeds that observed for artesunate, a drug that does not affect the rheological properties of circulating RBCs infected with ring-stage parasites (the only form usually found in the peripheral blood of patients with nonsevere falciparum malaria). Given the consequences of the altered deformability of infected RBCs on their normal function and survival in vivo [5, 10, 11, 15, 16], this feature reinforces the potential role of KAE609 as an important contributor to malaria control. The fact that it promotes clearance of RBCs infected with very young asexual ring-stage parasites, that are least sensitive to artemisinins, makes it a suitable drug partner in the fight to contain and possibly reverse the emergence of artesunate-tolerant parasites. Should additional studies indicate that it similarly promotes clearance of mature gametocytes, that would further raise its value as a transmission-blocking agent, a much sought-after characteristic in future drugs to be deployed for malaria elimination.

## Supplementary Data

Supplementary materials are available at http://jid.oxfordjournals.org. Consisting of data provided by the author to benefit the reader, the posted materials are not copyedited and are the sole responsibility of the author, so questions or comments should be addressed to the author.

Supplementary Data
