# Supplementary material for: A Basis for Rapid Clearance of Circulating Ring-Stage Malaria Parasites by the Spiroindolone KAE609
Source: J Infect Dis. 2015 Jul 1;213(1):100–4. doi: 10.1093/infdis/jiv358 (PMC4676544; doi:10.1093/infdis/jiv358)
Supplement: Supplementary Data [file supp_213_1_100__index.html]

A basis for rapid clearance of circulating ring-stage malaria parasites by the spiroindolone KAE609 — A Basis for Rapid Clearance of Circulating Ring-Stage Malaria Parasites by the Spiroindolone KAE609 — A Basis for Rapid Clearance of Circulating Ring-Stage Malaria Parasites by the Spiroindolone KAE609 — Supplementary Data 

# A Basis for Rapid Clearance of Circulating Ring-Stage Malaria Parasites by the Spiroindolone KAE609

## Supplementary Data

Supplementary Data

- Supplementary Data - Docx file
- Supplementary Video 1 - avi file
