# Supplementary material for: A Basis for Rapid Clearance of Circulating Ring-Stage Malaria Parasites by the Spiroindolone KAE609
Source: J Infect Dis. 2015 Jul 1;213(1):100–4. doi: 10.1093/infdis/jiv358 (PMC4676544; doi:10.1093/infdis/jiv358)
Supplement: Supplementary Data [file supp_jiv358_jiv358supp.docx]

**Supplemental Material**

**
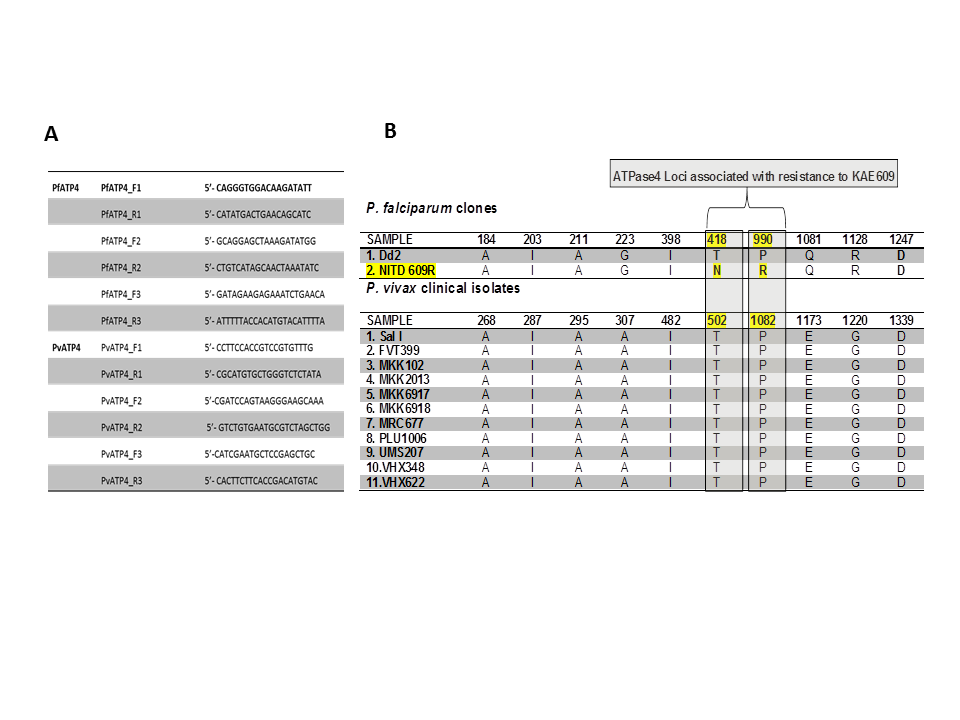
**

**Figure 1. (A)** The primers used to sequence PF3D7_1211900 (PfATP4) and PVX_084625 (PvATP4). PCR master mixtures were made with 200 µM of each dNTP, 0.5 µM forward primer, 0.5 µM reverse primer, 0.02 U/µL Phusion DNA polymerase, 6 µL of 5× Phusion HF buffer, and 1 µL of genomic DNA to a total reaction volume of 30 µl. Thermocycler parameters were as follows: 98°C for 30 s, followed by 35 cycles of 98°C for 10 s, 60°C for 30 s, and 72°C for 1 min. **(B)** The genotypes of *P. falciparum* PfATPase4 (PF3D7_1211900) and its ortholog; *P. vivax* ATPase4 (PVX_084625) in the two *P. falciparum* clones and ten isolates used in this study (The *P. vivax* SAL1 genotype was obtained from PlasmoDB [http://plasmodb.org]). Only two of the three mutations described by Spillman *et al* [[4](#_ENREF_4)] were observed in the genotype of ATPase4 from the spiroindolone resistant *P. falciparum* Dd2^R609^ (T418N and P990R). We did not detect any non-synonymous mutations in ATPase4 in the parasites from the 10 clinical isolates tested or in the KAE609 sensitive Dd2 *P. falciparum* clone.

**
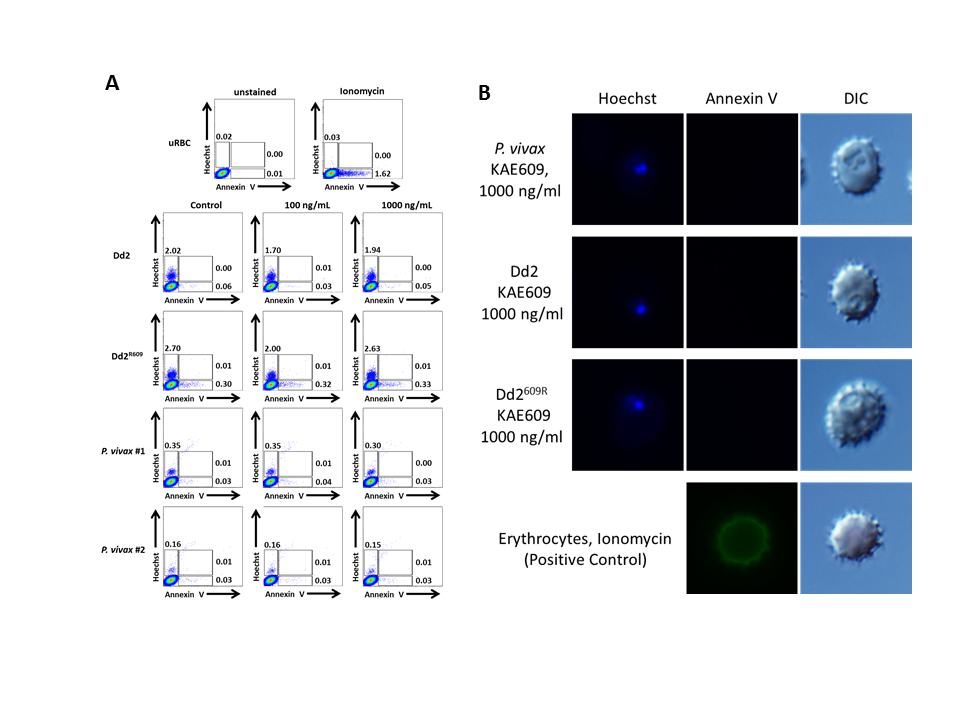
**

**Figure 2.** While high concentrations of KAE609 cause eryptotic changes to the infected host RBC (cell shrinkage and blebbing), they do not cause phosphatidylserine exposure. **(A)** Flow cytometry and **(B)** microscopic imaging of FITC Annexin V shows that 1000ng/mL (2 hours treatment) does not cause the flipping of phosphatidylserine (one of the key markers of eryptosis). Ionomycin was used as a positive control. It is important to note that the buffer specifically used for this assay causes the cells to become echinoid (an expected feature of this assay).

**Video 1. (Video)** Blockage of2µm microfluidic restrictions by RBCs infected with ring-stage *P. falciparum* (Dd2) treated with 100ng/mL of KAE609. KAE609 therapeutically disrupts normal deformability of RBCs infected with early stage parasites
